# Experimental Therapeutic Approaches for the Treatment of Retinal Pathology in Neuronal Ceroid Lipofuscinoses

**DOI:** 10.3389/fneur.2022.866983

**Published:** 2022-04-18

**Authors:** Udo Bartsch, Stephan Storch

**Affiliations:** ^1^Department of Ophthalmology, Experimental Ophthalmology, University Medical Center Hamburg-Eppendorf, Hamburg, Germany; ^2^University Children's Research@Kinder-UKE, University Medical Center Hamburg-Eppendorf, Hamburg, Germany

**Keywords:** neuronal ceroid lipofuscinoses, NCL, Batten disease, retinal degeneration, enzyme replacement therapy, gene therapy, lysosomal storage disorder

## Abstract

The neuronal ceroid lipofuscinoses (NCLs) are a group of childhood-onset neurodegenerative lysosomal storage disorders mainly affecting the brain and the retina. In the NCLs, disease-causing mutations in 13 different ceroid lipofuscinoses genes (CLN) have been identified. The clinical symptoms include seizures, progressive neurological decline, deterioration of motor and language skills, and dementia resulting in premature death. In addition, the deterioration and loss of vision caused by progressive retinal degeneration is another major hallmark of NCLs. To date, there is no curative therapy for the treatment of retinal degeneration and vision loss in patients with NCL. In this review, the key findings of different experimental approaches in NCL animal models aimed at attenuating progressive retinal degeneration and the decline in retinal function are discussed. Different approaches, including experimental enzyme replacement therapy, gene therapy, cell-based therapy, and immunomodulation therapy were evaluated and showed encouraging therapeutic benefits. Recent experimental ocular gene therapies in NCL animal models with soluble lysosomal enzyme deficiencies and transmembrane protein deficiencies have shown the strong potential of gene-based approaches to treat retinal dystrophies in NCLs. In CLN3 and CLN6 mouse models, an adeno-associated virus (AAV) vector-mediated delivery of *CLN3* and *CLN6* to bipolar cells has been shown to attenuate the retinal dysfunction. Therapeutic benefits of ocular enzyme replacement therapies were evaluated in CLN2 and CLN10 animal models. Since brain-targeted gene or enzyme replacement therapies will most likely not attenuate retinal neurodegeneration, there is an unmet need for treatment options additionally targeting the retina in patients with NCL. The long-term benefits of these therapeutic interventions aimed at attenuating retinal degeneration and vision loss in patients with NCL remain to be investigated in future clinical studies.

## Introduction

This review focuses on experimental approaches aimed at attenuating the progression of retinal degeneration in the different animal models of neuronal ceroid lipofuscinoses (NCLs). The NCLs are caused by defects in 13 different genes (CLN1–CLN8, CLN10–CLN14) encoding soluble lysosomal proteins (CLN1, CLN2, CLN5, CLN10, CLN11, and CLN13), membrane proteins located in the ER (CLN6 and CLN8), ER-Golgi intermediate compartment (CLN8) or lysosomes (CLN3, CLN7, and CLN12), and cytosolic proteins associated with synaptic vesicles (CLN4) or the plasma membrane (CLN14, Table 1) (1). The majority of affected gene products play important roles for lysosomal biogenesis and function: soluble lysosomal enzymes involved in lysosomal protein degradation (palmitoyl-protein thioesterase 1/PPT1, tripeptidyl-peptidase 1/TPP1, cathepsin D, and cathepsin F), soluble lysosomal proteins with unknown function (CLN5), and polytopic lysosomal membrane proteins (CLN3, CLN7, and CLN12). The primary function of the lysosomal membrane protein CLN3 is unclear (2). CLN7 was recently shown to function as an endolysosomal chloride channel (3). CLN6 and CLN8 form a complex in the endoplasmic reticulum (ER) which is required for the biosynthetic transport of a subset of newly synthesized soluble lysosomal proteins from the ER to the Golgi apparatus (4, 5). Biochemically, defects in NCL genes lead to lysosomal and autophagic dysfunction and subsequent accumulation of autofluorescent ceroid lipopigments. Based on the age of patients at the onset of first symptoms, the NCLs have been classified into congenital, infantile, late infantile, juvenile, and adult NCL phenotypes (6, 7). The clinical symptoms include seizures, progressive neurological decline, deterioration of motor and language skills, and dementia resulting in premature death (8). All disease-causing mutations and sequence variations in the *CLN* genes are summarized in the NCL mutation database (https://www.ucl.ac.uk/ncl-disease), and genotype-phenotype correlations are discussed in a recent review (9). With the exception of CLN4, CLN12, and CLN13 disease, deterioration and loss of vision is another major hallmark of NCLs (Table 1) (10). However, the deterioration of vision does not always appear as the first symptom in different NCLs (11). In rare cases, patients with CLN3 and CLN7 disease present with non-syndromic retina degeneration (12–14). To date, there are no curative therapies for the treatment of neurodegeneration in the brain and the retina, and patients rely on palliative treatment (15). An enzyme replacement therapy (ERT) using the intracerebroventricular infusions of recombinant TPP1 (cerliponase alfa) every 2 weeks has been shown to decelerate the disease progression and was recently approved for the treatment of patients with CLN2 disease (16).

**Table 1:** Summary of NCL forms, localization and function of gene products and retinal pathology in human patients.

**NCL form**	**Protein**	**Localization and function**	**Clinical phenotypes**	**Retinal pathology**
CLN1	Palmitoyl-protein thioesterase 1 (PPT1)	Lysosomal enzyme Long-chain fatty acyl hydrolase	Infantile^*^ Late infantile Juvenile Adult	Loss of vision, progressive retina atrophy, optic nerve atrophy
CLN2	Tripeptidyl-peptidase 1 (TPP1)	Lysosomal enzyme Serine protease	Late infantile^*^ Juvenile	Loss of vision, optic nerve atrophy
CLN3	CLN3	Lysosomal membrane protein Unknown	Juvenile^*^ Retinitis pigmentosa Adult Cone-rod dystrophy	Loss of vision is leading symptom, macular degeneration, optic nerve atrophy
CLN4	Cysteine string protein α	Cytoplasmic protein-associated with synaptic vesicles Regulation of neurotransmitter release, Exocytosis/endocytosis coupling	Adult^*^ (Autosomal-dominant, Parry disease)	Not known
CLN5	CLN5	Lysosomal protein Unknown	Late infantile^*^ Juvenile Adult	Retinal degeneration, loss of vision
CLN6	CLN6	ER membrane protein Biosynthetic transport from ER to Golgi	**Late infantile** ^*^ Adult	Loss of vision
CLN7	CLN7	Lysosomal membrane protein Chloride channel	Late infantile^*^ Juvenile Adult Macular dystrophy Cone-rod dystrophy	Loss of vision as later symptom
CLN8	CLN8	ER/ER-Golgi intermediate compartment membrane protein Biosynthetic transport from ER to Golgi	Late infantile^*^ Juvenile	Retinopathy and loss of vision, optic nerve atrophy
CLN10	Cathepsin D (CTSD)	Lysosomal enzyme Aspartic endoprotease	Congenital^*^ Late infantile Juvenile Adult	Congenital: unclear Retinopathy and loss of vision for later onset forms
CLN11	Progranulin (PGRN)	Lysosomal enzyme chaperone Neuronal survival and axonal growth factor	Adult^*^ Frontotemporal lobar dementia (heterozygous mutation)	Retinopathy and loss of vision
CLN12	ATP13A2	Lysosomal membrane protein Polyamine-transporting ATPase	Juvenile^*^	Not known
CLN13	Cathepsin F (CTSF)	Lysosomal enzyme Cysteine protease	Adult^*^ (Kufs disease)	Not known
CLN14	BTB/POZ domain-containing protein KCTD7 (KCTD7)	Cytoplasmic protein-partially associated with plasma membrane Unknown	Infantile Late infantile	Loss of vision, optic nerve atrophy

* *Phenotypes present in patients with a complete loss of protein function*.

Naturally occurring and gene-targeted mouse models and large animal models, such as dogs, sheep, and macaques, allowed the age-dependent morphological, biochemical, and functional analyses of retinal pathologies in NCL (17, 18). The pathomechanisms leading to the degeneration of neuronal cells in different retinal cell layers and the loss of retinal function are not well understood. CLN3 has been suggested to be required for the phagocytosis of photoreceptor outer segments by retinal pigment epithelial (RPE) cells (19). In line with this notion, lysosomal storage and increased numbers of mature autophagosomes and basal phagolysosomes were found in the retinal pigment epithelium of *Cln*3^Δ*ex*1−6^ mice, a CLN3 mouse model (20).

Preclinical studies targeting the brain of NCL animal models using ERT and gene therapy demonstrated a delayed onset and an attenuated progression of neuroinflammation and neurodegeneration (21). However, intracerebroventricular ERT and brain-targeted gene therapies are unlikely to prevent or attenuate neurodegeneration in the retina (15, 22). Therefore a combination of the brain- and eye-directed therapy might be required to prevent neurodegeneration in both the brain and the retina (23).

## Gene Therapy

Gene therapies for lysosomal storage disorders and NCLs are designed to correct the primary genetic defect (21). Experimental adeno-associated virus (AAV) vector-based brain-targeted gene therapies have been evaluated in animal models for CLN1, CLN2, CLN3, CLN5, CLN6, CLN7, CLN8, CLN10, and CLN11 disease (21, 24, 25). Based on the promising results of the preclinical studies, some of these brain-targeted gene therapies are currently being tested in clinical trials in CLN2, CLN3, and CLN6 patients (21). Ocular gene therapy is an emerging field. In general, gene therapies for soluble lysosomal enzyme deficiencies involve the cross-correction of non-transduced cells, and therapeutic benefits might thus be achieved with a relatively low number of successfully transduced cells. Gene therapies for membrane protein deficiencies, in comparison, will most likely require higher numbers of transduced cells to achieve therapeutic benefits (23). The most widely used viral vectors for ocular gene therapy are based on AAVs since they mediate stable, long-term transgene expression, and produce only minor immune responses (26). Preclinical ocular gene therapy studies in NCL animal models have mainly used intravitreally administered AAV vectors with different serotypes prior to the onset of the retinal pathology (27). Ocular gene therapy studies have been performed in animal models targeting soluble lysosomal protein deficiencies (CLN1, CLN5, CLN10, and CLN11) but also transmembrane protein defects (CLN3 and CLN6) (Table 2) and showed encouraging results. To date, the long-term therapeutic benefits of ocular gene therapy in human patients with NCL are unknown since no clinical trials are ongoing or have been completed (21).

**Table 2:** Preclinical ocular-targeted gene therapies for NCLs.

**NCL type**	**Animal model**	**Viral vector**	**Delivery route**	**Age of intervention**	**References**
CLN1	*Ppt1* knockout mouse	AAV2	Intravitreal	P18–21 or 8 weeks	(28)
CLN3	*Cln*3^Δ*ex*7/8^ knock-in mouse	AAV7m8	Intravitreal	P5 or P6	(29)
		AAV2/8	Subretinal	P14	(29)
CLN5	*CLN5*-deficient Borderdale sheep	AAV9	Intravitreal	3 months	(30)
CLN6	*Cln6/nclf* mouse	AAV7m8	Intravitreal	P5 or P6	(31)
	*CLN6*-deficient South Hampshire sheep	AAV9	Intravitreal	3 months	(30)
CLN10	*CtsD* knockout mouse	AAVshH10	Intravitreal	P5	(32)
CLN11	*Pgrn* knockout mouse	AAV2.7m8	Intravitreal	1, 6, and 12 months	(33)
		AAV9.2YF	Systemic	P3 or P4	

## CLN1 Disease

The CLN1 mouse model is characterized by the progressive loss of photoreceptors starting at 3 months of age and decreased retinal functions compared with wild type mice as measured by electroretinogram (ERG) recordings (28). Intravitreal administration of an AAV2 vector carrying human *PPT1* cDNA led to a five-fold increase in PPT1 enzymatic activities compared with age-matched wild type mice (28). Although a better organization of the photoreceptor layer and improved retinal function as measured by ERG recordings were detected in AAV2-*PPT1* treated mutant mice, the progression of the retinal dystrophy was only retarded but not completely prevented.

## CLN2 Disease

A naturally occurring *TPP1*-deficient Dachshund model recapitulates the key features of human CLN2 disease, including ataxia, tremor, progressive brain atrophy, loss of vision, and a reduced life span (34). For CLN2 disease, there are no reports on the efficacy of experimental ocular gene therapies in animal models. A single pre-symptomatic intraventricular injection of an AAV2 vector harboring canine *TPP1 (cTPP1)* cDNA into the CLN2 Dachshund model led to the reduced storage of autofluorescent material and decreased astrocytosis in the brain, and delayed onset of cognitive deficits and extended lifespan of the mutant dogs (35). However, TPP1 was not detected in photoreceptors and retinal pigment epithelial cells. In the treated CLN2 mutant dogs, the retinal degeneration and reduction of ERG b-wave amplitudes were not prevented by the brain-directed administration of AAV2-c*TPP1* compared with untreated dogs (36). These data suggest that AAV-mediated brain-targeted gene therapy is not sufficient to treat the retinal degeneration and loss of vision in CLN2 dog models.

## CLN3 Disease

The *Cln3* knock-in mouse model (*Cln*3^Δ*ex*7/8^) genetically recapitulates the 1 Kb deletion mutation of exons 7 and 8 found in 85% of human patients with CLN3 disease (37). Retinal degeneration in *Cln*3^Δ*ex*7/8^ mice is relatively mild leading to the loss of bipolar cells and a progressive reduction of the b-wave amplitudes beginning from 12 months of age (29, 38). These data demonstrate a progressive reduction of inner retinal function in the retinas of mutant mice. An ocular gene therapy using the intravitreal injections of an AAV7m8 vector harboring human *CLN3* in postnatal *Cln*3^Δ*ex*7/8^ mice led to the improved survival of bipolar cells and retinal function (29). In contrast, a subretinal injection using an AAV2/8-*mCln3* vector targeting photoreceptors and retinal pigment epithelial cells did not attenuate the loss of bipolar cells and the decline in inner retinal function suggesting that the expression of CLN3 in photoreceptors was not therapeutic in the mutant mice. Of note, the *Cln*3^Δ*ex*7/8^ mice do not fully recapitulate the retinal phenotype observed in patients with CLN3 disease. The number of photoreceptors was unchanged in the 15-month-old *Cln*3^Δ*ex*7/8^ mice whereas photoreceptors in the retinal postmortem tissues of patients with CLN3 disease are almost completely lost (7).

## CLN5 Disease

Murray and colleagues reported on the first successful intravitreal gene therapy in a large NCL animal model (30). They used the naturally occurring *CLN5*-deficient Borderdale sheep which recapitulates the key features of human CLN5 disease, such as motor and cognitive decline, progressive neurodegeneration in the brain and the retina, and loss of vision (39). The sheep eye represents a good model for the human eye because of its similar morphology and size. *CLN5*-deficient Borderdale sheep received a single intravitreal injection of an AAV9 vector harboring ovine *CLN5* cDNA at 3 months of age and were analyzed at 18 months of age (30). AAV9-*CLN5*-treated eyes showed minor lysosomal storage and neuroinflammation and intact retinal layers with a thickness comparable to that of the control sheep (30). In addition, the measurements of retinal functions in treated eyes showed ERG amplitudes nearly comparable with amplitudes in wild type control sheep (30).

## CLN6 Disease

The *Cln6* ^*nclf*^ mouse is a naturally occurring mouse model of CLN6 disease (40). The main pathological features in the retina of *Cln6* ^*nclf*^ mice include the loss of photoreceptors, early-onset reactive gliosis, accumulation of lysosomal storage material in multiple retinal cell layers, and the increased expression of soluble lysosomal enzymes (41). Surprisingly, an AAV2/8 vector-mediated gene transfer of human or mouse *CLN6* did not prevent the loss of photoreceptors and did not preserve photoreceptor functions in *Cln6* ^*nclf*^ *mice*. In contrast, an AAV2.7m8 vector-mediated bipolar cell-specific expression of *CLN6* prevented the loss of photoreceptors and preserved their function (31). Data indicated that *Cln6* deficiency in bipolar cells is the cause of photoreceptor degeneration in the *Cln6* ^*nclf*^ mouse. White and colleagues reported for the first time that brain-targeted gene therapy in *Cln6* ^*nclf*^ mice attenuated retinal pathology. Intracerebroventricular gene therapy in *Cln6* ^*nclf*^ mice reduced the pathology in visual centers of the brain and in the retina (42). The intracerebroventricular injection of an AAV9-CBV-*CLN6* vector into postnatal day 1 (P1) *Cln6* ^*nclf*^ mice reduced the degeneration of photoreceptors in 3-, 6-, and 9-month-old mice compared with untreated controls. In striking contrast, a brain-directed AAV-mediated expression of cathepsin D in a CLN10 mouse model prevented the accumulation of ceroid lipofuscin, the activation of microglia, and neurodegeneration in brain tissues, but not the rapidly progressing retinal degeneration (43). Intravitreal injection of an AAV9 vector encoding *CLN6* into a naturally occurring *CLN6*-deficient South Hampshire sheep model led to a minor reduction of lysosomal storage and retinal atrophy and had no beneficial effects on retinal function as indicated by unaltered ERG amplitudes compared with untreated contralateral eyes (30).

## CLN10 Disease

The CLN10 mouse deficient in the lysosomal protease cathepsin D (CTSD) is a model for the most severe NCL form, congenital NCL. Cathepsin D knockout (*Ctsd* ko) mice are characterized by an early-onset loss of photoreceptor cells and a subsequent loss of all other retinal nerve cell types, the accumulation of storage material, lysosomal dysfunction, reduced autophagic flux, reactive astrogliosis and microgliosis, and a shortened lifespan with premature death at P26 (44, 45). The *Ctsd* ko mouse is a valuable model to study the efficacy of experimental ocular therapies due to the early-onset and most rapid progression of retinal degeneration in all NCL forms. Intravitreal administration of an AAVsh10 vector harboring mouse *Ctsd* transduced retinal glial cells and RPE cells (32). Biochemical and morphological analyses of the AAV-treated retinas revealed a restoration of CTSD enzymatic activities close to wild type levels, a complete reduction of lysosomal storage material, the absence of lysosomal hypertrophy, and the preservation of photoreceptor and rod bipolar cells. However, this gene therapy study was unable to clarify whether the retinal function was preserved due to ethical issues related to experiments on severely affected animals at the end stage of the disease.

## CLN11 Disease

In a mouse model for CLN11 disease, the progranulin knockout (*Pgrn* ko) mouse, autofluorescent storage material accumulation, and the degeneration of photoreceptors and retinal ganglion cells become apparent in 12-month-old mutant mice (46). In a recent study, the therapeutic benefits of intravenous administration of an AAV9.2YF-*Pgrn* vector were compared with the intravitreal delivery of an AAV2.7m8-*Pgrn* vector into *Pgrn* ko mice (33). Systemically administered AAV9 vectors cross the blood-retina- and blood-brain-barriers until 7 days of age. Intravenous delivery of an AAV9.2YF vector encoding murine *PGRN* into P3 or P4 *Pgrn* ko mice led to a reduction of autofluorescent ceroid lipopigments and attenuated the thinning of the outer nuclear layer and the total retina in 12-month-old mutant mice (33). Interestingly, intravitreal injection of an AAV2.7m8-*Pgrn* vector into 1- or 6-month-old *Pgrn* ko mice reduced lipofuscin lipopigments, decreased microglial infiltration, but did not attenuate retinal neurodegeneration. These data suggest that both the route and time of AAV administration are crucial to achieving therapeutic benefits in the retina of *Pgrn* ko mice.

## Enzyme Replacement Therapy

Ocular enzyme replacement therapies (ERTs) rely on the intravitreal administration of a recombinant soluble lysosomal enzyme and its uptake *via* mannose 6-phosphate receptors, delivery to lysosomes, and cross-correction (47). Therapeutic benefits of ocular ERTs have been tested in animal models for CLN2 and CLN10 diseases (15).

Periodic intravitreal injections of recombinant TPP1 starting at 12 weeks of age into the CLN2 Dachshund dog model led to decreased neurodegeneration in the inner nuclear layer and inhibited declines in ERG amplitudes (48). Intravitreal TPP1 administration also prevented focal retinal detachments in the mutant dogs. A single intravitreal injection of recombinant CTSD into P7 and P14 CLN10 mutant mice partially attenuated lysosomal dysfunction and reduced reactive microgliosis but failed to prevent the photoreceptor loss and retinal degeneration (49). The data suggest that the regular intravitreal administration of a recombinant lysosomal enzyme may be a therapeutic option to treat retinal degeneration and vision loss at least in some NCL forms. A new clinical trial (Clinical Trial gov Identifier: NCT05152914) is currently enrolling patients to test the therapeutic efficacy of intravitreal ERT (Cerliponase alfa) in CLN2 disease.

## Cell-Based Therapy

Therapeutic benefits of cell-based ERTs were evaluated in NCL animal models (15). Cell transplantation into the retina of NCL models deficient in soluble lysosomal proteins is based on the rationale that the grafted cells secrete the missing lysosomal proteins (donor cells) followed by their internalization *via* mannose 6-phosphate receptors by surrounding defective acceptor cells (47). The lysosomal enzyme-mannose 6-phosphate receptor complexes are internalized and lysosomal enzymes are finally targeted to lysosomes where they are proteolytically activated. Preclinical experiments evaluated the benefits of transplanted stem cells overexpressing the missing lysosomal enzyme. Stem cells transduced with an AAV2-vector carrying the human *PPT1* cDNA were intravitreally implanted at early disease stages into the CLN2 Dachshund model (50). A single injection of these modified stem cells inhibited the pathological changes in retinal morphology and retinal function suggesting that genetically modified stem cells might serve as useful vehicles for a long-term intraocular administration of the soluble lysosomal protein in NCLs. In another approach, neural stem cells that were transduced *ex vivo* with a lentiviral vector harboring murine *Ctsd* cDNA were intravitreally implanted into the CLN10 mouse model of the CLN10 mouse model (32). In treated retinas, the restoration of CTSD enzymatic activities to 44% of wild type levels, a partial decrease of lysosomal storage material, and reduced microgliosis and astrocytosis compared with untreated *Ctsd* knockout retinas were detected. However, the degeneration of different retinal cell types was not prevented by the implanted stem cells. In summary, the data suggest that intravitreal injection of genetically modified stem cells may be an encouraging approach to attenuate retinal degeneration for some NCL forms with soluble lysosomal enzyme deficiencies.

## Immunomodulation

Previous studies showed that the genetic inhibition of the adaptive or innate immune system led to disease-ameliorating effects in the CNS of *Ppt1* ko/*Cln*3^Δ*ex*1−6^ ko and *Ppt1* ko mice, respectively (51, 52). In line with these findings, the treatment of *Ppt1* ko or *Cln*3^Δ*ex*1−6^ ko mice with immunosuppressive drugs showed therapeutic benefits (53, 54). Oral administration of the immunomodulators fingolimod and teriflunomide prevented retinal thinning in *Ppt1* ko mice and attenuated retinal thinning in *Cln*3^Δ*ex*1−6^ ko mice (55). In a genetically modified *Cln*3^Δ*ex*7/8^ knock-in mouse susceptible to light damage, light exposure resulted in pathological changes, including retinal neurodegeneration, activation of microglia, and accumulation of autofluorescent storage material (56). Treatment of the mutant mice with the antibiotic and anti-inflammatory drug minocycline prior to light stress led to reduced photoreceptor loss and decreased amounts of autofluorescent storage material (56). Based on the strong reactive gliosis present in *Cln6* ^*nclf*^ retinas, mutant mice were treated with the natural immunomodulators curcumin and docosahexanoic acid [DHA, (57)]. In the curcumin- and DHA-treated *Cln6* ^*nclf*^ mice, reactive gliosis was attenuated and the decline in visual acuity and ERG amplitudes was delayed when compared with untreated mutant mice.

## Discussion

Retinal degeneration and loss of vision are among the major hallmarks of NCLs of NCLs. With the exception of one study on *Cln6* ^*nclf*^ mice, brain-targeted therapies in NCL animal models had no therapeutic impact on retinal degeneration and loss of retinal function. Therefore, there is an unmet need to design novel eye-targeted therapies. The therapeutic efficacy of eye-targeted experimental therapies in NCL animal models, including gene therapy, enzyme replacement therapy, cell-based therapy, and immunomodulation, were evaluated in the past. Recent experimental ocular gene therapies on animal models with soluble lysosomal enzyme deficiencies (CLN1, CLN5, CLN10, and CLN11) and transmembrane protein deficiencies (CLN3 and CLN6) have shown the strong potential of gene therapeutic approaches to effectively treat NCL-related retinopathies. A major breakthrough in the experimental gene therapy approaches was the identification of the specific cell types that have to be targeted to achieve therapeutic benefit. In the CLN3 and CLN6 mouse models, the AAV-mediated bipolar cell-specific delivery of *CLN3* and *CLN6* was successful in preventing the loss of photoreceptors and bipolar cells, respectively, and to partly preserve retinal function. Furthermore, the intravitreal injection of an AAV9-*CLN5* vector into a CLN5 sheep model largely prevented retinal degeneration and loss of retinal function. Finally, results from a recent study suggest that an AAV-mediated CTSD expression in the retina of a CLN10 mouse model is more potent in preventing retinal degeneration than intravitreal ERT mediated by the injections of recombinant CTSD or by transplantation of neural stem cells overexpressing CTSD. While the results of the eye-targeted therapies are encouraging, most studies have started the treatment prior to the onset of the retinal pathology. Future work thus needs to evaluate whether the treatment strategies are still effective when they are started at the initial or advanced stages of the retinal dystrophy. For clinical applications, gene therapies have the advantage of single dosing compared with enzyme replacement therapies which require repeated administration. Combined therapies targeting the brain and the retina separately may attenuate neurological symptoms and additionally vision loss in patients with NCL. The long-term benefits of these experimental ocular treatment options have to be evaluated in patients with NCL in future clinical studies.

## Author Contributions

UB and SS interpreted the data and wrote the review. Both authors contributed to the article and approved the submitted version.

## Funding

The laboratory works of the authors were supported by funding from the Mila's Miracle Foundation (SS), the Stiftung zur Förderung der Universitätsmedizin Hamburg, and the Ernst and Berta Grimmke Stiftung (UB).

## Conflict of Interest

The authors declare that the research was conducted in the absence of any commercial or financial relationships that could be construed as a potential conflict of interest.

## Publisher's Note

All claims expressed in this article are solely those of the authors and do not necessarily represent those of their affiliated organizations, or those of the publisher, the editors and the reviewers. Any product that may be evaluated in this article, or claim that may be made by its manufacturer, is not guaranteed or endorsed by the publisher.
